# A Decade of Pediatric CA-MRSA Surveillance in Northern Taiwan: Retrospective Resistance Analysis and Recent Genotypic Characterization

**DOI:** 10.3390/microorganisms13051013

**Published:** 2025-04-28

**Authors:** Chia-Ning Chang, Chia-Hsiang Yu, Chih-Chien Wang

**Affiliations:** Department of Pediatrics, Tri-Service General Hospital, National Defense Medical Center, Taipei 114202, Taiwan; lizy0529@hotmail.com (C.-N.C.); phil790419@gmail.com (C.-H.Y.)

**Keywords:** MRSA, SCCmec, children, clindamycin resistance, co-trimoxazole, genotyping

## Abstract

Methicillin-resistant *Staphylococcus aureus* (MRSA) is a major cause of pediatric infections and has shown evolving molecular characteristics over time. This study aimed to investigate the phenotypic and genotypic features of MRSA isolates collected from pediatric patients at a tertiary medical center in northern Taiwan between 2011 and 2020. A total of 182 MRSA strains were analyzed for SCCmec types, PVL gene presence, antimicrobial susceptibility, multilocus sequence typing (MLST), and clonal relatedness using pulsed-field gel electrophoresis (PFGE). ST59/SCCmec Vt was the most prevalent genotype, followed by ST59/SCCmec IV and ST8/SCCmec IV. Most ST59/SCCmec Vt and ST8/SCCmec IV isolates clustered genetically. Clindamycin and erythromycin resistance remained high, whereas co-trimoxazole susceptibility ranged from 76% to 100%. These findings confirm ST59 as the dominant clone and highlight the emergence of ST8 and ST45 in community-associated MRSA (CA-MRSA) infections. Oral co-trimoxazole remains the most effective empirical option, while clindamycin and erythromycin should be avoided. Continuous molecular surveillance is warranted to monitor trends and guide treatment strategies in pediatric MRSA infections.

## 1. Introduction

*Staphylococcus aureus*, a notorious microbe, has extensively colonized and been transmitted across communities and healthcare institutions for more than a century. The epidemiology of methicillin-resistant *Staphylococcus aureus* (MRSA) and its associated antibiotic resistance has remained a significant global issue for over 60 years. MRSA causes a wide spectrum of diseases ranging from minor skin lesions to life-threatening sepsis. The selection of effective antibiotics and appropriate clinical management is often challenging [1].

In particular, community-acquired MRSA (CA-MRSA) often carries Panton–Valentine Leukocidin (PVL), a virulence factor that can destroy epithelial cells and leukocytes, contributing to the development of severe infections. Treatment failure and increasing mortality have been reported among pediatric patients with invasive MRSA infections, including bacteremia, endocarditis, septic arthritis, osteomyelitis, and meningitis. MRSA serves as both a commensal colonizer and a clinical pathogen. Colonization raises the rate of infection, since infecting strains match colonizing strains in as many as 50–80% of cases [2]. In our previous study, the nasal MRSA colonization among children in northern Taiwan increased from 8.1% (2004–2006) to 15.1% (2007–2009) [3]. Nevertheless, the incidence of MRSA nasal colonization did not increase in central Taiwan in 2005–2010 but decreased in the prevalence of MSSA (methicillin-sensitive *Staphylococcus aureus*) nasal colonization [3]. In the United States, although invasive MRSA infections have declined since 2005, an epidemiological shift toward more virulent methicillin-sensitive *S. aureus* (MSSA) infections has been reported in children. Sutter et al. analyzed susceptibility trends in pediatric *S. aureus* isolates for over a decade, revealing a modest increase in oxacillin susceptibility and a slight decline in clindamycin susceptibility. Notably, virulence determinants were not assessed in their study [4]. A similar temporal shift has occurred in Taiwan. After an initial decline, MRSA infections in children without known risk factors have markedly increased. A 2011 study reported that over 50% of pediatric *S. aureus* infections in northern Taiwan were MRSA, with high resistance to clindamycin (>90%), erythromycin (>90%), and chloramphenicol (57–65%), as well as lower resistance to gentamicin (21–34%) [5]. In 2015, another research in northern Taiwan found that MRSA resistances were “erythromycin (41.3%) clindamycin (38%), chloramphenicol (20.6%), and gentamicin (17.2%)”; co-resistance to erythromycin and clindamycin was also noted for 38% of MRSA isolates [6]. In addition, a recent report revealed that clinical MRSA isolates from 2008 to 2018 showed a statistically significant increase in clindamycin susceptibility (CLI-S) from 4.9% in 2008 to 33.2% in 2018 (Ptrend < 0.001) [7].

MRSA strains are highly diverse in their molecular characteristics. Specific genotypes such as ST59, ST8 (USA300), and ST45 have emerged as predominant clones in different geographic regions, each associated with varying antimicrobial susceptibility and virulence. In Taiwan, ST59 has long been recognized as the dominant CA-MRSA lineage, while ST8 (USA300) has recently gained clinical importance due to its rapid dissemination and association with severe infections. ST45, although less common, is increasingly reported in long-term care facilities and among colonized individuals. Therefore, genotype-based surveillance is critical for tracking MRSA evolution, identifying emerging clones, and informing regional infection control policies. As highlighted above, pediatric MRSA strains in Taiwan show dynamic temporal and molecular changes, underscoring the need for continuous epidemiological surveillance. The aim of this study was to characterize MRSA isolates from pediatric patients both phenotypically (antibiotic susceptibility) and genotypically (MLST and SCCmec typing), and to assess their genetic relatedness through dendrogram-based clustering.

## 2. Materials and Methods

### 2.1. Study Design and Clinical MRSA Isolates

This retrospective study was conducted from 2011 to 2020 at a 1400-bed tertiary medical center in northern Taiwan. All patients were under the age of 18 years old and hospitalized with an MRSA infection identified from medical records and the Bacteria Room of the Clinical Pathology Department. The study protocol was approved by the Institutional Review Board of Tri-Service General Hospital in Taipei, Taiwan (Permit number: TSGHIRB No:2-106-05-059)

During the retrospective 10-year study period, a total of 182 MRSA strains were isolated altogether. The definition of CA-MRSA infection was as with any MRSA infection diagnosed within 48 h of admission to hospital, of which the patient has none of the following risk factors for healthcare-associated MRSA infection, such as residence at a nursing home or hospital during the prior year, presence of an enduring catheter or percutaneous instrument at the time of culture, or previous isolation of MRSA.

### 2.2. DNA Extraction

Extraction of chromosomal DNA from all collected *S. aureus* strains was performed by the pure gene DNA purification kit (Gentra Corporation, Minneapolis, MN, USA). The main steps are briefly described as follows: 1. Take the amount of bacteria in a ring, add 400 μL of deionized water, and break up the *S. aureus* colonies by shaking. 2. Add 3 μL of lysostaphin and then invert it upside down to mix well, then place it in a 37 °C water bath for one hour. 3. Add 300 μL of cell lysate and mix well by rapid shaking, then place in a dry bath at 80 °C for 15 min. 4. Remove the above sample and let it cool at room temperature. Add 3 μL of ribonuclease and then invert it upside down to mix well. Then put it in a 37 °C water bath for 15–30 min. 5. After cooling the above sample to room temperature, add another 100 μL of protein precipitation solution, and then oscillate at high speed for 20 s. 6. Centrifuge at 13,000 rpm for 3 min, remove the supernatant, then add 500 μL of isopropanol and turn it upside down for about 50 times to mix well. 7. Centrifuge at 13,000 rpm for 1 min and then remove the supernatant. Add 600 μL of 70% alcohol and turn it upside down for about ten times to mix well. 8. Centrifuge again at 13,000 rpm for 1 min and carefully pour the supernatant. 9. Perform an air-drying step to completely remove the remaining 70% alcohol. 10. If there is a white precipitate at the bottom, add 200 μL of DNA hydration solution; if no white precipitate is present at the bottom, add only 100 μL of DNA hydration solution. 11. Place it in a water bath at 65 °C for one hour or overnight at room temperature to dissolve the extracted DNA evenly. 12. Determine and adjust the concentration of DNA to 10–20 ng/mL. All DNA samples are then stored in a freezer at −80 °C for subsequent experiments.

### 2.3. Genotyping

Chromosomal DNAs were extracted from MRSA strains for molecular characterizations. The MRSA isolates were analyzed by pulsed-field gel electrophoresis (PFGE) with staphylococcal cassette chromosome (SCCmec) typing via the multiplex PCR method, and the detection of Panton Valentine Leukocidin (PVL) genes was achieved by using a PCR assay. Representative strains were further selected for multilocus sequence typing (MLST) and pulsotyping. PFGE was performed via using the CHEF Mapper XA system (BioRad Laboratories, Hercules, CA, USA) and we designated the pulsotypes in alphabetical order. For the purpose of PFGE polymorphisms recognition, band patterns were identified using Molecular Analyst Fingerprinting Software, version 1.6 (Bio-Rad Laboratories, Hercules, CA, USA). Grouping was performed to produce a dendrogram from the matrix using the unweighted pair group method with the arithmetic averages setting technique after estimation of similarities by using the Pearson correlation coefficient between each pair of organisms; the PFGE patterns were distinguished at the 80% similarity level. SCCmec typing was classified by using a multiplex polymerase chain reaction with sets of region-specific primers as formerly portrayed.

In this study, 128 out of the 182 MRSA isolates were classified as community-associated MRSA (CA-MRSA) strains, based on the clinical definitions described earlier. All CA-MRSA isolates underwent SCCmec typing and PVL gene detection.

Multilocus sequence typing (MLST) and pulsed-field gel electrophoresis (PFGE) were performed on a subset of 50 representative CA-MRSA isolates collected between 2018 and 2020. These isolates were selected based on the availability of viable stored strains and their representativeness in terms of dominant SCCmec types and infection sites. PFGE was used to assess clonal relatedness among the isolates, while MLST was applied to identify the sequence types associated with each SCCmec subtype.

### 2.4. Antimicrobial Susceptibility Testing

A total of three antimicrobial agents—clindamycin, erythromycin, and co-trimoxazole—were tested for antimicrobial susceptibility based on the hospital’s routine practice during the study period. Laboratory records of susceptibility data for all MRSA strains were reviewed, and duplicate isolates from the same patient were excluded from the analysis. MRSA identification and antimicrobial susceptibility testing were conducted in accordance with the Clinical and Laboratory Standards Institute (CLSI) guidelines [8]. Due to the retrospective nature of the study, susceptibility data for other key agents such as vancomycin and linezolid were not available; this limitation has been acknowledged in the Discussion section.

### 2.5. Statistical Analysis

All data were entered into Microsoft Excel 365 (Microsoft Corporation, Redmond, WA, USA) and analyzed using the Data Analysis Tool Pak in Excel. Comparisons of the distributions of dichotomous and continuous variables were performed with the Chi-square test and Student’s *t* test, respectively. *p*-Value of <0.05 were considered significant.

## 3. Results

During the ten-year study period (2011 to 2020), a total of 182 MRSA strains were isolated from pediatric patients under the age of 18 years old and were obtained from the Bacteria Room of the Clinical Pathology Department. Analyzing these 182 MRSA strains by the SCCmec types, we divided them into 25 strains of type II, 29 strains of type III, 72 strains of type IV, and 56 strains of type Vt; details of molecular characterizations including PVL genes detection for these 182 isolates are shown in Table 1. Furthermore, skin and soft tissue infection showcased the greatest number of infection sites with 85 strains (46.7%), followed by 62 strains of respiratory infection (34%), 34 strains of sterile site infection (including blood, CSF, urine, pleural fluid, synovial fluid) (18.7%), as revealed in Table 2. To investigate temporal trends in antimicrobial resistance, we stratified the data into two periods: 2011–2015 and 2016–2020. As shown in Table 3A,B, susceptibility of clindamycin and erythromycin remained low among SCCmec III and Vt isolates across both periods, whereas co-trimoxazole consistently exhibited high susceptibility across all SCCmec subtypes.

**Figure 1:**
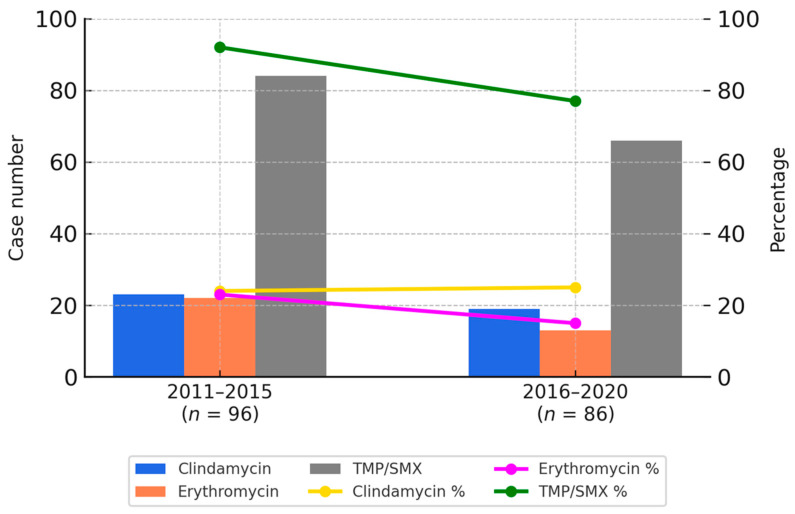
Bar charts represent the number of resistant CA-MRSA isolates to clindamycin (blue), erythromycin (orange), and TMP/SMX (gray) during two study periods. Line graphs depict the corresponding susceptibility percentages of each antibiotic: clindamycin (yellow), erythromycin (purple), and TMP/SMX (green). TMP/SMX showed the highest susceptibility rates in both periods.

Overall, the susceptibility rates for clindamycin and erythromycin were approximately 20% in both periods. In contrast, co-trimoxazole showed a higher susceptibility rate, although a modest decline was observed—from 86% in the first period to 76% in the second. These temporal trends are illustrated in Figure 1.

Figure 1 provides a graphical representation of the antimicrobial susceptibility trends between 2011 and 2015 as well as 2016 and 2020. Bar charts indicate the number of resistant isolates for each antibiotic, while the line graphs reflect the respective susceptibility percentages. These visualizations allow for a clear comparison of temporal changes in resistance patterns, consistent with the data presented in Table 3A,B.

Furthermore, Table 3 summarizes the antimicrobial susceptibility patterns by SCCmec subtype, demonstrating that co-trimoxazole maintained excellent in vitro activity, with susceptibility rates reaching up to 96% among certain subtypes. The variation in susceptibility across SCCmec types was statistically significant (χ^2^ = 23.5671, *p* = 0.000627; *p* < 0.05).

Among the 182 MRSA isolates, 128 (70%) were classified as CA-MRSA. Among these, 28% of SCCmec IV strains (20/72) and 93% of SCCmec Vt strains (52/56) were PVL-positive. In terms of infection site distribution among CA-MRSA strains, skin and soft tissue infections (SSTIs) were the most common (72/128, 56.3%), followed by respiratory infections (39/128, 30.5%).

Of the 50 representative CA-MRSA isolates genotyped, the most frequent sequence types were ST59/SCCmec Vt (18 isolates, 36%), followed by ST59/SCCmec IV (10 isolates, 20%), ST45/SCCmec IV (7 isolates, 14%), and ST8/SCCmec IV (7 isolates, 14%).

**Figure 2:**
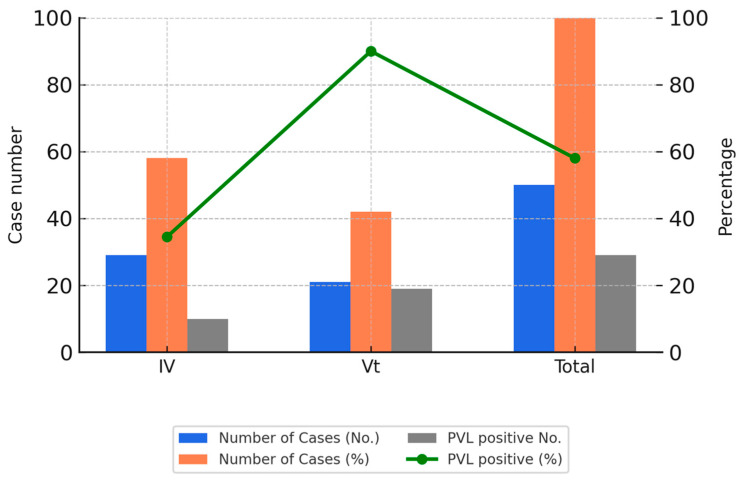
SCCmec subtypes and PVL gene positivity among 50 CA-MRSA isolates. Bar charts display the number and percentage of CA-MRSA isolates carrying SCCmec type IV or Vt, along with the number of PVL-positive isolates. The line graph shows the PVL gene positivity rate in each group. SCCmec Vt demonstrated the highest PVL positivity rate, suggesting a shift in molecular epidemiology.

These isolates were collected between 2018 and 2020 and were selected based on sample availability and the feasibility of extended molecular testing. The selection aimed to reflect recent trends and allowed for in-depth analysis of clonal distribution. Figure 2 illustrates the PVL positivity rates among the SCCmec IV and Vt subtypes in these genotyped isolates.

**Figure 3:**
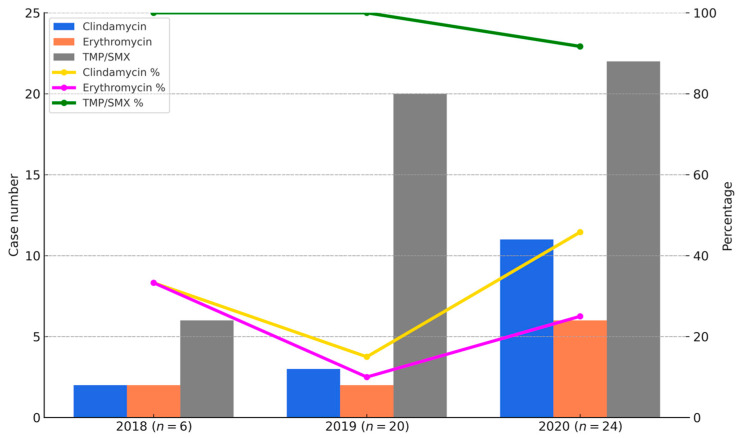
Annual resistance and susceptibility trends of CA-MRSA isolates to clindamycin, erythromycin, and TMP/SMX from 2018 to 2020. Bar charts indicate the number of CA-MRSA isolates resistant to clindamycin (blue), erythromycin (orange), and TMP/SMX (gray). Line graphs show the susceptibility percentages for the same antibiotics: clindamycin (yellow), erythromycin (purple), and TMP/SMX (green). TMP/SMX demonstrated the highest and most stable susceptibility, while clindamycin and erythromycin showed lower and more variable trends.

As shown in Figure 3, the bar graphs represent the number of CA-MRSA isolates resistant to clindamycin, erythromycin, and co-trimoxazole in each year (2018–2020), while the line graphs indicate the corresponding susceptibility rates. Resistance to erythromycin and clindamycin remained high throughout the study period, with susceptibility rates of only 10% and 15% in 2018, increasing to 40% and 50% in 2020, respectively. In contrast, susceptibility to TMP-SMX remained consistently high, with rates of 100% in 2018, 95% in 2019, and 91.7% in 2020. Figure 3 provides a visual summary of annual changes in CA-MRSA resistance between 2018 and 2020. However, because the distribution of SCCmec subtypes varied across these years, the resistance data should be interpreted descriptively, rather than as evidence of statistically validated temporal trends.

As shown in Table 4, ST59 was the most common sequence type among SCCmec IV isolates (10/29), followed by ST8 and ST30. Although ST59 was even more prevalent among SCCmec Vt isolates (18/21), its presence within SCCmec IV subtypes reflects the historical dominance of the ST59/SCCmec IV lineage in Taiwan’s CA-MRSA profile.

**Figure 4:**
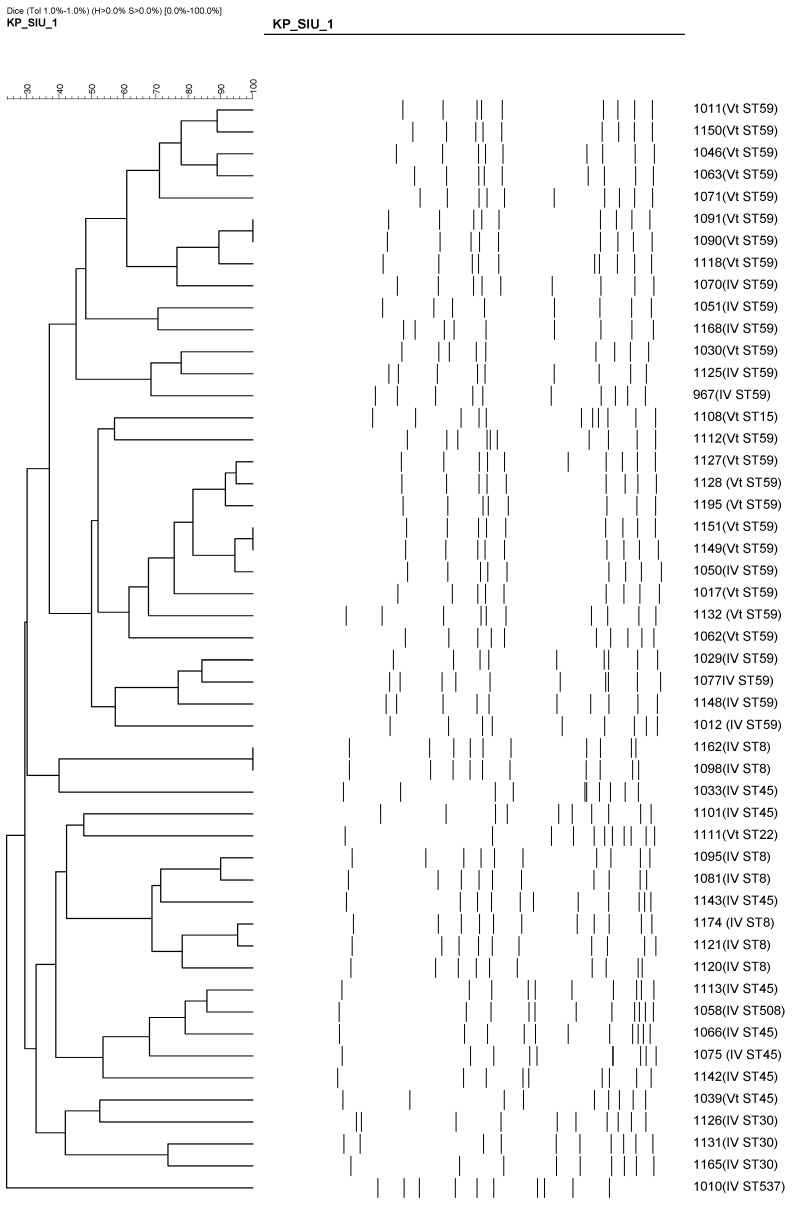
PFGE-based dendrogram showing the clonal relationships among 50 CA-MRSA isolates. Clustering patterns are based on >80% similarity. Most ST59/SCCmec Vt and ST8/SCCmec IV isolates grouped into distinct clonal clusters, suggesting local transmission and clonal expansion of specific MRSA lineages.

In contrast, SCCmec Vt was strongly associated with emerging clones, including ST59 (18/21), ST45 (8/21), and ST15/22. Notably, most of these Vt isolates were PVL-positive. The clustering of these isolates based on PFGE results is shown in Figure 4, highlighting the genetic diversity among the Vt clones. This trend suggests a shift in the molecular epidemiology of pediatric CA-MRSA, marked by the increasing emergence of virulent and genetically diverse clones beyond the traditional ST59/SCCmec IV combination.

In addition to SCCmec typing, we further analyzed the antimicrobial susceptibility profiles according to MLST sequence types among the 50 CA-MRSA isolates collected between 2018 and 2020. The detailed distribution of resistance to clindamycin, erythromycin, and trimethoprim-sulfamethoxazole (TMP/SMX) among different sequence types is summarized in Supplementary Table S1. This analysis highlights variations in resistance patterns between traditional clones (such as ST59/SCCmec IV) and emerging clones (such as ST59/SCCmec Vt and ST45/SCCmec Vt).

## 4. Discussion

The antimicrobial resistance of *methicillin-resistant Staphylococcus aureus* (MRSA) has become an urgent public health concern. MRSA is a leading cause of infections in both community and healthcare settings and is increasingly demonstrating resistance to multiple antimicrobial agents worldwide.

This hospital-based 10-year surveillance study provides detailed clinical insights into pediatric MRSA infections in northern Taiwan. We found that community-associated MRSA (CA-MRSA) isolates exhibited high susceptibility to co-trimoxazole but showed high resistance rates to erythromycin and clindamycin [7]. In particular, ST59 strains demonstrated the highest resistance rates to clindamycin (85.7%) and erythromycin (82.1%), whereas ST8 strains showed comparatively lower resistance (57.1% and 71.4%, respectively). These findings suggest clonal differences in antimicrobial resistance profiles among circulating CA-MRSA lineages.

A similar trend has been reported outside of Taiwan. A study from Israel found that clindamycin resistance among community-associated *S. aureus* strains was notably high, underscoring the challenge of selecting appropriate empirical therapy for pediatric skin and soft tissue infections [9].

Serious MRSA infections have also become increasingly difficult to manage due to the widespread emergence of multidrug resistance mechanisms, which have significantly limited effective therapeutic options in many clinical settings [10].

Understanding local epidemiology is therefore essential to guide appropriate empirical therapy for suspected *S. aureus* infections. In our study, the clindamycin resistance rate among MRSA isolates ranged from 63.0% to 91.7%, based on the susceptibility patterns of different SCCmec types. Specifically, resistance was highest in SCCmec Vt (95%) and lowest in SCCmec II (64%). Consistent with our data, Lee et al. reported that in 2017, clindamycin resistance rates reached 66.2% in custodial facilities and 73.1% in a regional hospital in central Taiwan [11]. In contrast, Yueh et al. analyzed pediatric skin and soft tissue infections in northern Taiwan and observed a dynamic trend in clindamycin susceptibility: 22.9% in 2010, declining to 5.7% in 2013, then increasing to 61.1% in 2019 [12]. These results highlight the regional and temporal variability of clindamycin resistance in MRSA and underscore the importance of continuous local antimicrobial surveillance. These findings collectively emphasize the importance of ongoing regional antimicrobial resistance surveillance to guide empirical treatment strategies for MRSA infections. Additionally, in our advanced studies, the MRSA resistance rate to clindamycin even reached 90.7% during 2004–2007 [3]. After analyzing antimicrobial susceptibility for methicillin-resistant Staphylococcus aureus (MRSA) colonizing isolates in Taiwanese children through 2004–2009, the MRSA resistance rates to clindamycin were 87.9% and 89.6% in the health maintenance visits group and kindergartens group, respectively [3]. Data adapted from Lo et al. [3]. As mentioned above, most CA-MRSA were highly resistant to clindamycin and erythromycin in Taiwan [6], and antibiotic susceptibility patterns of CA-MRSA in a recent study were also compatible with the aforesaid results [12,13]. Physicians should be aware of regional susceptibility patterns when choosing empirical regimens. Clindamycin alone is not an ideal choice for children with invasive MRSA infections in Taiwan. In addition, a contemporaneous study from mainland China reported a clindamycin resistance rate of 86.8% among CA-MRSA isolates collected from pediatric respiratory tract samples between 2015 and 2020 [13].

Of note, the main infection site was skin and soft tissue infection (SSTI) (85 isolates, 46.7%). On the other hand, about 70.3% (128 strains) belonged to CA-MRSA. Since 2000, the incidence of skin and soft tissue infections (SSTIs) has risen substantially, along with associated hospitalizations and medical expenditures, largely due to the widespread emergence of community-associated methicillin-resistant *Staphylococcus aureus* (CA-MRSA) [14,15]. Traditionally, clinicians used antimicrobial susceptibility profiles to differentiate community-associated from healthcare-associated MRSA. However, this approach has become increasingly unreliable, as many CA-MRSA strains now exhibit resistance to non-β-lactam antibiotics [2]. A multicenter randomized trial demonstrated that both clindamycin and trimethoprim-sulfamethoxazole (TMP-SMX) were effective in treating uncomplicated skin and soft tissue infections (SSTIs), with comparable clinical cure rates. However, clindamycin showed modest advantages in pediatric subgroups and in cases involving abscesses [16].

In our investigation, *S. aureus* isolates demonstrated varying levels of susceptibility to co-trimoxazole, with rates ranging from 45.4% to 91.6% depending on SCCmec type. These findings suggest that, in regions or clinical settings where *S. aureus* susceptibility to co-trimoxazole remains high, this agent may be considered as one of the empiric treatment options for mild to moderate community-associated SSTIs in pediatric patients. However, in areas where resistance exceeds 50%, its empirical use should be avoided in favor of agents with higher efficacy.

In summary, while studies from the United States have documented a significant shift in the epidemiology of pediatric *S. aureus* infections over the past two decades [14,15], such trends have not been prominently observed in Taiwan, based on both current and historical data.

This longitudinal study also highlighted recent changes in the molecular epidemiology of MRSA among pediatric patients at a medical center during a three-year period (2018–2020). The most frequently identified genotype was ST59/SCCmec Vt (18 strains, 36%), followed by ST59/SCCmec IV (10 strains, 20%), ST45/SCCmec IV (7 strains, 14%), and ST8/SCCmec IV (7 strains, 14%). Previous studies have reported that three MRSA sequence types—ST59, ST8, and ST45—are commonly circulating in the community. ST59 has been the predominant CA-MRSA clone in Taiwan, while ST8 (USA300) and ST45 have emerged more recently, with increasing reports of their involvement in both community and hospital infections [17,18]. In contrast to ST59, ST8 (USA300) has historically been uncommon in Taiwan. However, recent data have shown a rising prevalence of ST8 MRSA in both community and healthcare settings. Huang et al. [17] reported that approximately 13% of USA300 isolates were classified as hospital-onset, suggesting that this lineage may be expanding its clinical footprint in Taiwan. When comparing our findings with previous studies, we carefully distinguished whether their MRSA isolates represented CA-MRSA, HA-MRSA, or mixed sources to ensure appropriate contextualization of the molecular and resistance trends we observed.

An epidemic as well as an endemic community clone, ST59/SCCmec Vt, dominated throughout the study period [19]. In addition to ST59, ST45 MRSA has been increasingly reported as a prevalent strain, particularly in nursing homes and long-term care facilities in Taiwan [20,21]. Studies have shown that ST45 can account for up to 40–50% of MRSA colonization among residents in certain LTCFs [22]. In our current study, ST45/SCCmec IV (7 strains; 14%) was the second most common genotype following ST59. ST8/SCCmec IV was also identified in 7 strains (14%). This observation aligns with recent studies indicating that ST8 (USA300) has been circulating in Taiwan for more than a decade. Huang et al. reported that the prevalence of ST8 MRSA increased from less than 3% before 2016 to approximately 16–20% in more recent years, depending on the clinical setting and population studied [17,23]. In our data, the proportion of ST8/SCCmec IV was 14%, which is consistent with these earlier reports. Huang et al. also noted that nearly 13% of USA300 isolates from bloodstream or pediatric samples were categorized as hospital-onset, identified from specimens collected more than 48 h after admission [17]. Similarly, Peng et al. found that ST8/SCCmec IV strains (presumably USA300) accounted for 16.3% (36 out of 221) of MRSA isolates obtained from patients with cellulitis and osteomyelitis at a regional hospital in southern Taiwan between 2016 and 2018 [23].

In our study, 73 out of 182 MRSA isolates (40%) were PVL-positive. The majority of these PVL-positive strains were found among SCCmec type Vt (93%, 52/56), followed by type IV (27%, 20/72), and a single case among type III (3%, 1/29). None of the SCCmec type II isolates carried the PVL gene. These findings are consistent with recent reports that emphasize the dynamic nature of MRSA epidemiology in pediatric settings [24,25,26]. It is essential that physicians refer to current microbiological surveillance data from their own institutions, as the distribution of MRSA clones and resistance patterns can vary significantly between hospitals and over time [24,25]. As antimicrobial resistance becomes increasingly widespread, the risk of invasive MRSA infections in pediatric patients rises accordingly [26].

In terms of clonal clustering, recent studies have shown that distinct MRSA lineages are closely associated with specific antibiotic resistance profiles and virulence factors [27]. This highlights the growing importance of infection prevention and control strategies within community settings, which must be informed by ongoing molecular surveillance and whole-genome analysis of circulating MRSA clones [27].

Several potential limitations should be acknowledged in this study. First, the overall sample size of MRSA isolates was relatively limited, which may reduce the statistical power and hinder generalization of the findings to the broader pediatric population in northern Taiwan. However, since this study was conducted in a major tertiary medical center—Tri-Service General Hospital (TSGH)—where the majority of patients likely reside in the eastern Taipei metropolitan area, the data may still reflect important regional trends.

Second, whole-genome sequencing (WGS) data for the MRSA isolates were not available, which limits the resolution of genotypic and evolutionary analyses. The interpretation of strain diversity thus relied on SCCmec typing, PVL gene detection, and partial MLST/PFGE data.

Third, molecular genotyping and dendrogram analysis were performed on only 50 representative CA-MRSA isolates. This was primarily due to the limited availability of archived strains; many isolates collected prior to 2018 were no longer viable or had not been preserved. Furthermore, because of resource constraints, extended genotyping (PFGE and MLST) was limited to isolates collected during 2018–2020. These 50 strains were selected to represent the most recent and epidemiologically relevant samples, allowing meaningful interpretation of clonal diversity while acknowledging this limitation.

Fourth, antimicrobial susceptibility testing was limited to only three antibiotics—clindamycin, erythromycin, and co-trimoxazole—due to retrospective data constraints. During the study period, the hospital microbiology laboratory routinely performed susceptibility testing only for these agents, as they were the most commonly prescribed agents for pediatric community-associated MRSA infections. This selection reflected institutional practices, pediatric prescribing guidelines, and national surveillance priorities at the time. As a result, data for key agents such as vancomycin and linezolid were not systematically collected or retrievable from the laboratory information system. This limitation may reduce the comprehensiveness of resistance surveillance and limit the generalizability of our findings to all therapeutic contexts.

These limitations, while inherent to the study’s retrospective design and resource availability, underscore the need for prospective studies incorporating larger sample sizes, full-genome sequencing, and broader antibiotic panels to enhance the generalizability and precision of CA-MRSA surveillance in pediatric populations.

## 5. Conclusions

In conclusion, MRSA isolates in our pediatric cohort exhibited high susceptibility to co-trimoxazole (91.7–100%), but markedly lower susceptibility to clindamycin and erythromycin. For mild to moderate community-associated MRSA (CA-MRSA) infections in children, empirical treatment should prioritize co-trimoxazole, whereas clindamycin and erythromycin may not be appropriate due to high resistance rates.

In terms of molecular epidemiology, ST59, ST8 (USA300), and ST45 have emerged as the major community MRSA clones in Taiwan. The increasing detection of ST8 and ST45 highlights the need for continued molecular surveillance to monitor clonal shifts and assess their clinical impact.

Although molecular characterization in this study was limited to isolates collected up to 2020, the trends observed provide an important baseline for understanding the evolution of CA-MRSA in the pediatric population. Future prospective studies are warranted to determine whether these patterns have persisted or changed in the post-pandemic era.

## Figures and Tables

**Table 1:** SCCmec types and PVL gene status among 182 MRSA isolates collected from pediatric patients (2011–2020). The table presents the number and percentage of isolates with each SCCmec type and the presence or absence of the PVL gene.

SCCmec Subtypes	II	III	IV	Vt	Total
Number of Cases (%)	25 (14%)	29 (16%)	72 (40%)	56 (30%)	182 (100%)
PVL-positive No. (%)	0 (0%)	1 (3%)	20 (27%)	52 (93%)	73 (40%)

**Table 2:** Distribution of infection sites among MRSA isolates. Skin and soft tissue infections (SSTIs) were the most common presentation, followed by respiratory and sterile site infections.

	SCCmec II	SCCmec III	SCCmec IV	SCCmec Vt	MRSA(%)
Respiratory infections	12	11	31	8	62 (34%)
Skin and soft tissue infections	9	4	35	37	85 (46.7%)
Sterile site infections	4	14	5	11	34 (18.7%)
Other infection	0	0	1	0	1 (0.6%)
Total	25	29	72	56	182 (100%)

**Table 3:** Antimicrobial susceptibility of MRSA isolates stratified by SCCmec subtype. The table shows susceptibility rates for clindamycin, erythromycin, and co-trimoxazole across different SCCmec groups. Chi-square test results are included to assess significance between groups. (A). Antibiotic susceptibility of MRSA isolates by SCCmec type (2011–2015, *n* = 96). (B). Antibiotic susceptibility of MRSA isolates by SCCmec type (2016–2020, *n* = 86).

(A)			
SCCmec Type	Clindamycin S/Total (%)	Erythromycin S/Total (%)	Co-Trimoxazole S/Total (%)
II	4/10 (40%)	4/10 (40%)	9/10 (90%)
III	1/10 (10%)	1/10 (10%)	8/10 (80%)
IV	16/46 (35%)	16/46 (35%)	39/46 (85%)
Vt	2/30 (7%)	1/30 (3%)	24/30 (80%)
(B)			
SCCmec Type	Clindamycin S/Total (%)	Erythromycin S/Total (%)	Co-Trimoxazole S/Total (%)
II	4/15 (27%)	2/15 (13%)	14/15 (93%)
III	1/19 (5%)	1/19 (5%)	3/19 (16%)
IV	6/26 (23%)	5/26 (19%)	23/26 (88%)
Vt	0/26 (0%)	0/26 (0%)	25/26 (96%)

**Table 4:** Genotypic characteristics of 50 CA-MRSA isolates collected between 2018 and 2020 includes sequence type (ST), SCCmec type, PVL gene presence, and antimicrobial susceptibility profiles.

ST Type	Number (%)	SCCmec IV	SCCmec Vt
8	7 (14%)	7	0
15	1 (2%)	0	1
22	1 (2%)	0	1
30	3 (6%)	3	0
45	8 (16%)	7	1
59	28 (56%)	10	18
508	1 (2%)	1	0
573	1 (2%)	1	0
Total	50 (100%)	29	21

## Data Availability

The original contributions presented in this study are included in the article/Supplementary Material. Further inquiries can be directed to the corresponding author.

## Supplementary Materials

The following supporting information can be downloaded at: https://www.mdpi.com/article/10.3390/microorganisms13051013/s1, Table S1: Antimicrobial Susceptibility by MLST Type (2018–2020).
