# Supplementary material for: A Decade of Pediatric CA-MRSA Surveillance in Northern Taiwan: Retrospective Resistance Analysis and Recent Genotypic Characterization
Source: Microorganisms. 2025 Apr 28;13(5):1013. doi: 10.3390/microorganisms13051013 (PMC12114221; doi:10.3390/microorganisms13051013)
Supplement: Supplementary file 1 [file microorganisms-13-01013-s001.zip › microorganisms-3564208-supplementary.pdf]

Supplementary Table S1. Antimicrobial Susceptibility by MLST Type (2018–2020)

This table summarizes the antimicrobial susceptibility profiles of the 50 CA-MRSA isolates genotyped by MLST between 2018 and 2020. The table includes resistance counts and percentages for clindamycin, erythromycin, and co-trimoxazole by sequence type.

MLST Type	No. of Isolates	Clindamycin Resistance (%)	Erythromycin Resistance (%)	TMP-SMX Resistance (%)
ST59	28	85.7%	82.1%	7.1%
ST8	7	57.1%	71.4%	0%
ST45	8	75.0%	62.5%	0%
Others	7	28.6%	42.9%	0%
